# Lighting up PNETs: Creating Murine Models with a Novel Bioluminescent Cell Line

**DOI:** 10.1245/s10434-026-19089-z

**Published:** 2026-02-03

**Authors:** Matthew C. Moccia, Rachel Nation, T. Hess, Gena V. Topper, Ami Kalola, Michael Wang, Hannah Sofield, Zena Saleh, Xiaofeng Zhao, Yahui Li, Francis Spitz, Tao Gao, Young Ki Hong

**Affiliations:** 1 Department of Surgery, Cooper University Health Care, Camden, NJ USA; 2 Department of Pathology, Cooper University Health Care, Camden, NJ USA; 3 Department of Surgery, Rowan University School of Medicine, Camden, NJ USA; 4 Camden Cancer Research Center, Camden, NJ USA; 5 MD Anderson Cancer Center at Cooper, Camden, NJ USA

**Keywords:** Pancreatic neuroendocrine tumor, Orthotopic pancreatic implantation, Bioluminescence live imaging, Lineage tracing

## Abstract

**Background:**

Pancreatic neuroendocrine tumors (PNETs) are rare malignancies with limited treatment options beyond surgery, particularly in advanced stages, highlighting the need for novel therapeutic strategies. Featured by pronounced biologic heterogeneity, PNETs are driven by dysregulation of complex molecular-signaling pathways during tumor progression. This underscores the importance of biologically relevant and cost-effective preclinical models that allow convenient real-time monitoring of tumor progression and tracking of individual tumor cells, a current gap in the field.

**Methods:**

To address this challenge, the authors engineered a novel enteroendocrine tumor-derived STC-1 murine cell line that stably co-expresses firefly luciferase (F-Luc) and enhanced green fluorescent protein (EGFP). These dual-labeled cells were implanted into immunocompromised mice via three different routes: subcutaneous (SubQ), renal capsule (RC), and orthotopic pancreatic (OP) injections. Tumor growth was tracked using bioluminescence in vivo imaging, and PNET characteristics were assessed by histologic, immunohistochemical (IHC) and co-immunofluorescence analyses.

**Results:**

Tumors formed in all three models. The SubQ model showed rapid tumor growth but lacked key PNET features based on hematoxylin and eosin (H&E) and IHC staining. The RC model exhibited moderate growth but limited expression of PNET-specific markers. In contrast, the OP model demonstrated robust tumor growth and most closely resembled well-differentiated, grade 1 human PNETs. Lineage-tracing experiments further exhibited clonal architecture and dynamic progression within the OP model.

**Conclusions:**

Orthotopic pancreatic implantation of dual-labeled STC-1 cells provides a biologically relevant and pragmatic preclinical platform that closely resembles human PNETs. This model offers valuable utility for investigating PNET biology and evaluating novel therapeutic strategies.

**Supplementary Information:**

The online version contains supplementary material available at 10.1245/s10434-026-19089-z.

Pancreatic neuroendocrine tumors (PNETs) are rare malignancies, with an estimated incidence of 0.32 per 100,000 individuals.^1,2^ These tumors present significant clinical challenges due to the poorly understood molecular mechanisms that underpin their development and progression.^3–8^

The molecular pathogenesis of PNETs is intricate and characterized by complex interactions among numerous signaling pathways. Each pathway plays a role in different facets of tumor development, encompassing cell proliferation, survival, migration, and angiogenesis. Together, these pathways constitute a complex network that, when disrupted, can result in uncontrolled tumor growth and metastasis.^9–15^

Despite advancements in understanding the molecular landscape of PNETs, current therapeutic strategies remain largely ineffective, particularly in advanced-stage disease. Chemotherapy and targeted therapies have yielded some success, yet the high degree of molecular heterogeneity and intrinsic complexity of these tumors present substantial barriers to effective treatment.^16–20^ These challenges underscore the critical need for novel therapeutic approaches. Central to this effort is the development and utilization of robust preclinical models capable of accurately evaluating the efficacy of emerging therapeutic agents, especially for patients with advanced-stage PNETs, for which current treatment options are scarce.^19,21–23^

Various tumor models have been developed in cancer research, including tumor-derived cell lines, three-dimensional organoids, genetically engineered mouse models (GEMMs), patient-derived xenografts (PDXs), and tumor cell line-derived xenografts (CDXs).^18,24–28^ Among these, the CDX models remain widely used due to their ease of manipulation, low maintenance requirements, and rapid turnaround for evaluating therapeutic efficacy. However, their clinical relevance can be limited, particularly in recapitulating the complex tumor biology observed in patients.^29^ In addition, establishing PNET-specific xenograft models presents further obstacles. Only a limited number of PNET cell lines are available for research, and even fewer murine-derived lines, which are particularly useful for making syngeneic mouse xenograft models for studying tumor-immune interactions and immunotherapy development. These limitations are largely due to the low incidence and indolent growth of PNETs compared with other major tumor types.^30^ In addition, the pronounced heterogeneity of PNETs, which includes diverse genetic mutations and a complex cellular composition of tumor, stromal, and immune cells, makes it difficult to monitor the behavior of individual cell populations during tumor progression.^17,31^ Moreover, although subcutaneous xenografts are easy to monitor, other commonly used xenograft models, such as intrasplenic, intrarenal, or orthotopic pancreatic (OP) implantations, are more challenging to track non-invasively in real time, thereby limiting their application in PNET progression and drug development studies.^10,32–34^

To address these limitations, the current study aimed to develop a bioluminescent- and fluorescent-traceable PNET model using the intestinal secretin tumor (STC-1) cell line, which shares key morphologic and functional characteristics with human PNET cells, thereby making it a biologically relevant and translationally significant model system.^32,35,36^ This novel system enables real-time monitoring of tumor progression using in vivo imaging (IVIS) and facilitates ex vivo lineage-tracing of tumor cells in harvested tissues. To demonstrate its utility in PNET research, we systematically compared PNET tumor growth across three commonly used implantation methods, including subcutaneous, renal capsule, and OP injections,^37^ and assessed their biologic relevance by evaluating histopathologic features and tumor marker expression in comparison with human PNET specimens. Our work provides a valuable tool for PNET research and establishes a robust preclinical model for evaluating novel therapeutic strategies.

## Methods

### Development of an STC-1 Cell Line that Stably Expresses Firefly Luciferase and Enhanced Green Fluorescent Protein (EGFP) in Tandem

Murine-derived STC-1 cells (Cat# CRL-3254, ATCC) were plated at a density of 1.5 × 10^5^ cells per well in a 6-well plate and cultured until 70% confluence was reached. Lentiviral particles that encode a fusion peptide containing an N-terminal firefly luciferase, a viral 2A linker, and a C-terminal EGFP protein (Cat# LP464-025, GeneCopoeia) were added to cell culture in the presence of 4 µg/mL polybrene for 10 h. At 5 days after transduction, cells with 2 µg/mL of puromycin were selected, determined through a killing curve experiment. After puromycin selection, the cells were allowed to grow for 1 month and subsequently underwent fluorescence-activated cell-sorting (FACS) for further purification and expansion.

### Surgical Implantation of STC-1 PNET Cells

Transduced STC-1 cells were injected into immunocompromised Nu/J mice (Jackson Laboratory), which were divided into three experimental groups (4 per group) based on the anatomic injection sites. In brief, the mice were anesthetized with 2.5% isoflurane during procedures. A total of 1 × 10^6^ cells in 50 µL of medium were mixed with 50 µL of Matrigel and implanted into the subcutaneous space (SubQ), under the renal capsule (RC) or into the pancreas (OP), as previously described.^25,33,34^ An additional control group was established with 50 µL of Matrigel and 50 µL of PBS injected into mouse pancreas. The mice were maintained, and experimental procedures were performed in strict accordance with the guidelines approved by the Cooper Institutional Animal Care and Use Committee (IACUC #23-022). Throughout the experimental period, the mice were monitored on a weekly basis for body weight, grooming behavior, and overall activity levels, and no systemic toxicity was observed.

### In Vivo Bioluminescence Imaging

Tumor growth was monitored weekly up to 6 weeks using the Spectral In Vivo Imaging System (IVIS) for non-invasive bioluminescent imaging. At 10 min before each imaging, tumor-bearing mice were intraperitoneally injected with 200 µL of D-luciferin (15 mg/mL). Bioluminescence images were captured by IVIS and analyzed by defining regions of interest (ROI) to quantify tumor burden.

### Histologic and Immunohistochemical Analysis

At the conclusion of each experiment, tumors and distant organs were harvested for assessment of tumor burden and metastasis. Formalin-fixed, paraffin-embedded tissue blocks were sectioned at 5 μm and mounted onto slides for histologic and immunohistochemical (IHC) analysis.^21^ Hematoxylin and eosin (H&E) staining confirmed tumor presence and morphology. Immunohistochemistry was performed to detect neuroendocrine markers using the following antibodies: Ki-67 (Abcam, ab15580, 1:1000), synaptophysin (Cell Signaling, 36406, 1:100), chromogranin A (ThermoFisher, PA5-77917, 1:500), secretin (Bioss, bs-0088R, 1:200), insulin (Proteintech, 15848-1-AP, 1:500), pancreatic polypeptide (Proteintech, 15493-1-AP, 1:1000), and GFP (Abcam, ab290, 1:1000). Secondary anti-rabbit antibody was applied at a 1:200 dilution ratio. Negative controls were included by substituting primary antibodies with phosphate-buffered saline.

For comparative analysis, human well-differentiated PNETs and adjacent non-tumor tissues (Cooper University Hospital IRB #23-130) were included. The density and intensity of IHC-staining in PNET and adjacent benign tissue sections were quantified using the ImageJ software, which allows for the measurement of pixel intensity within specific regions of interest, providing an objective analysis of protein expression levels across samples. To ensure rigorous pathologic assessment, a board-certified pathologist was involved in characterizing tumor architecture and validating the models against established criteria for neuroendocrine differentiation.

### Statistical Analysis

Statistical comparisons between experimental groups were performed using ANOVA in GraphPad Prism 10. Results are reported as mean differences (MDs) with corresponding *p* values. A *p* value lower than 0.05 was considered statistically significant.

## Results

### Development of Bioluminescence/Fluorescence Reporter PNET Models

**Fig. 1:**
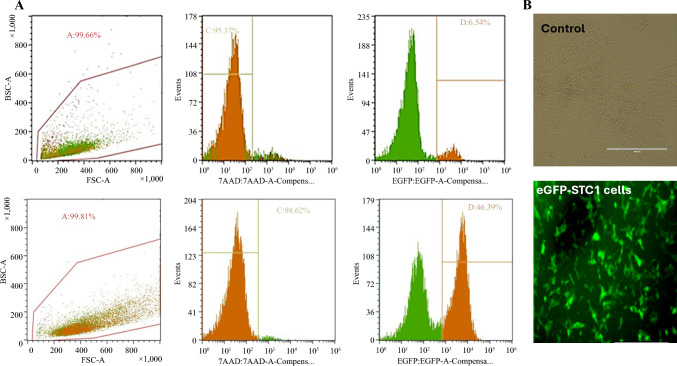
Characterization of enhanced green fluorescent protein (EGFP) transduction efficiency and in vivo tumor imaging. **A** Flow cytometric analysis of cell viability and transduction efficiency. Representative dot and histogram plots depict forward scatter (FSC-A) versus side scatter (SSC-A) gating (*left panels*), 7-AAD staining for viability (*middle panels*), and EGFP expression for transduction efficiency (*right panels*). The top row represents the control condition, whereas the bottom row shows the experimental group. Gated populations indicate high viability (>95%) and demonstrate an increase in EGFP-positive cells from 6.54% in the control group to 46.39% in the experimental group, reflecting successful transduction. Percentages within each plot denote the proportion of cells within the defined gate. **B** Phase-contrast microscopy (*left*) and fluorescence microscopy (*right*) of cells after transduction with an EGFP-expressing vector. The control condition (*top panel*) shows non-transduced cells with no detectable fluorescence, whereas the transduced population (*bottom panel*) exhibits robust green fluorescence, indicating successful gene transfer and EGFP expression. Scale bar = 400 µm

As shown in Fig. 1A, STC-1 cells, transduced with lentiviral vector encoding firefly luciferase and EGFP, were subjected to puromycin selection and further purified by FACS The transduced STC-1 cells expressed the transgene stably longer than 6 months, as indicated by the constitutive expression of EGFP. No significant changes in cell morphology or growth characteristics were observed, indicating that transduction did not adversely affect cell biology (Fig. 1B).

**Fig. 2:**
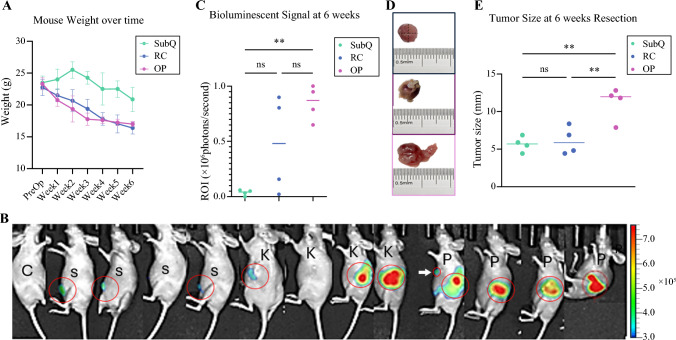
Tumor growth and mouse weight dynamics in a subcutaneous-kidney-pancreas model. **A** Mouse weight over time, documented weekly before surgery, with the mean and standard error shown for the subcutaneous-kidney-pancreas model. Data represent the weight changes across the study period. **B** At 6 weeks after implantation, bioluminescence imaging was performed to show signal localization in subcutaneous (S), kidney (K), and pancreatic (P) regions in mice injected with luciferase-expressing STC-1 cells. Control mice (C) show no detectable signal. One animal in the orthotopic pancreatic (OP) model demonstrated evidence of metastatic spread to the pancreas, indicated by an isolated focal signal distinct from primary implantation sites (*white arrow*). Bioluminescence imaging was performed using spectral in vivo imaging (IVIS). Heatmap scale represents radiance (photons/sec/cm^2^/sr). **C** Bioluminescent intensity of regions of interest (ROI) across the three experimental groups was recorded as photons per second and analyzed using one-way ANOVA test. **D** Tumor size measurements were taken *ex vivo* at the time the mice were killed as an indication of tumor progression. Note: The renal capsule tumor was embedded into the kidney organ for evaluation. **E** Tumor size was measured and analyzed by ANOVA test 6 weeks after inoculation. Statistical significance is indicated as *(*p* < 0.05), **(*p* < 0.01), and ns (not significant)

After successful injection of STC-1 cells in to the Nu/J mice, we first determined the overall growth condition of the three STC-1 xenograft models. As anticipated, all three experimental groups of STC-1 xenograft mice experienced weight loss during the 6-week study period. Notably, weight loss was significantly greater in the RC and OP tumor models than in the SubQ tumor model (Fig. 2A and Table S1).

To further evaluate the effect of tumor burden on overall growth condition, we performed a two-way ANOVA test to assess the effects of time and tumor type on mouse body weight. The analysis showed significant main effects for the time factor (F[6,63] = 24.23; *p* < 0.0001), the tumor model factor (F[2,63] = 90.24; *p* < 0.0001), and the interaction between time and tumor model (F[12,63] = 3.142; *p* = 0.0015). For the SubQ tumor model, the weight of the mice did not show a significant decrease between the preoperative period and week 6 (*p* > 0.05). In contrast, the mice in the RC tumor model showed modest weight loss from the preoperative period to weeks 3, 4, 5, and 6 (*p* < 0.05). The OP tumor model demonstrated significant weight loss from the preoperative period to weeks 2, 3, 4, 5, and 6 (*p* < 0.01), suggesting the more profound physiologic impact of tumor burden of OP PNET models (Table S1).

Meanwhile, bioluminescence imaging (BLI) was used to monitor tumor initiation and progression. By 6 weeks, all three models exhibited detectable signals, indicating aggressive tumor growth. Notably, one animal in the OP group exhibited metastatic dissemination (Fig. 2B), and the OP tumors showed significantly higher BLI signals within the ROI compared with SubQ tumors (Fig. 2C).

The mice were killed 6 weeks after implantation, and tumors were harvested for size measurement. The tumors in the SubQ and RC models exhibited slower growth, with mean sizes of 5.78 mm and 6.23 mm, respectively. In contrast, the OP tumors were significantly larger, averaging 11.33 mm (*p* < 0.01 vs SubQ and RC), indicating more aggressive growth (Fig. 2D and E). These findings align with the BLI results, validating bioluminescence imaging as a reliable indicator of tumor progression.

### Tumors Arising from OP Implantation of STC-1 Cells Recapitulate Grade 1 Human PNETs.

To assess the tumor identity of the three xenograft models, we conducted comprehensive histologic and IHC analyses. The tumors were harvested 6 weeks after implantation and analyzed by both H&E staining and IHC staining. Grades 1 and 2 human PNETs were used as histologic and IHC references. We first compared the histopathologic features of STC-1 cell-derived tumors with human PNETs using H&E staining.

**Fig. 3:**
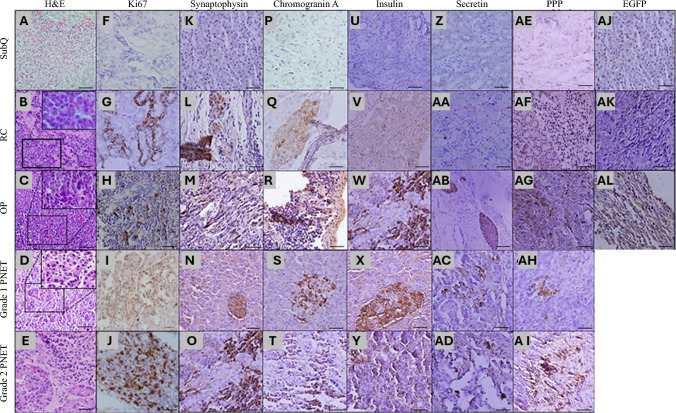
Histologic and immunohistochemical analysis of murine xenograft tumors in comparison with human grades 1 and 2 pancreatic neuroendocrine tumors (PNETs). Murine tumors generated via subcutaneous, kidney capsule, and orthotopic pancreatic implantation were compared with human grades 1 and 2 PNETs. (**A–E**) Tumor sections were analyzed by hematoxylin and eosin (H&E) staining and immunohistochemistry for (**F–J**) Ki67, (**K–O**) synaptophysin, (**P–T**) chromogranin A, (**U–Y**) insulin, (**Z–AD**) secretin, (**AE–AI**) pancreatic polypeptide (PPP), and (**AJ–AL**) enhanced green fluorescent protein (EGFP). Immunohistochemical (IHC) markers were used to assess proliferation, neuroendocrine differentiation, and hormone expression, whereas EGFP staining identified implanted STC-1cells in tumors of all three xenograft models. Human PNETs served as histologic and IHC references for comparison. Scale bar = 200 µm

**Fig. 4:**
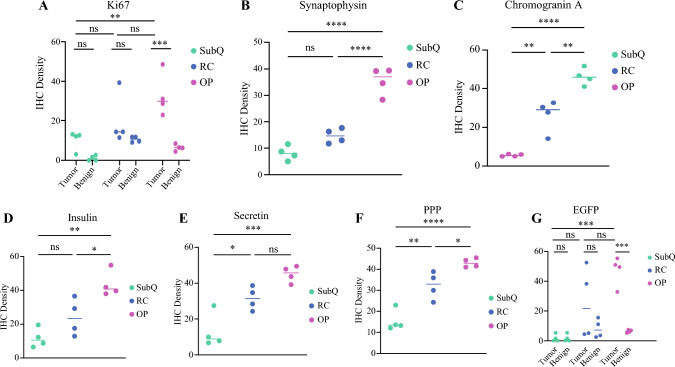
Immunohistochemical (IHC) marker-density comparison between tumor models. The density of IHC neuroendocrine markers (e.g., Ki67, synaptophysin, chromogranin A, insulin, secretin, pancreatic polypeptide [PPP], and enhanced green fluorescent protein [EGFP]) was quantified and compared between tumors of SubQ, RC, and OP models. Statistical analysis was performed using ANOVA with Tukey’s post hoc test. A dot plot was created for graphical/visual comparison of the ANOVA data. Statistical significance is indicated as *(*p* < 0.05), **(*p* < 0.01) , ***(*p* < 0.001), ****(*p* < 0.0001), and ns (not significant)

Among the three mouse PNET models, both RC and OP tumors displayed morphologic features characteristic of grade 1 human PNET (Fig. 3A–E and insets of Fig. 3B–D). In addition, PNET cell proliferation was assessed via Ki67-staining, which showed significantly lower Ki67 expression in SubQ tumors than in OP tumors (MD, 22.53; adjusted *p* = 0.0043). However, the difference between SubQ and RC tumors was not statistically significant (MD; 9.620, adjusted *p* = 0.4551). Each tumor model was compared with adjacent benign tissue, and only OP tumors showed a statistically significant difference (MD, 26.43; adjusted *p* = 0.0009). Pathologic assessment identified Ki67 expression in OP tumors as grade 1, consistent with human PNET grade 1 classification (Figs. 3F–J and 4A).

Furthermore, we evaluated the expression of well-established neuroendocrine markers.^38–40^ Synaptophysin expression was significantly higher in OP tumors than in either SubQ (MD, 27.2; adjusted *p* < 0.0001) or RC (MD, 20.68; adjusted *p* < 0.0001) tumors. No significant difference was detected between SubQ and RC tumors (MD, 6.516, adjusted *p* = 0.0844) (Figs. 3K–M and 4B). Similarly, chromogranin A expression was significantly higher in OP tumors than in either SubQ (MD, 40.46; adjusted *p* < 0.0001) or RC (MD, 20.64; adjusted *p* = 0.0012) tumors. Moreover, OP tumors exhibited a significant increase in chromogranin A expression relative to RC tumors (MD, 19.82; *p* = 0.0016) (Figs. 3P–R and 4C), indicating robust neuroendocrine differentiation in tumors of the OP model. Insulin expression was markedly higher in OP tumors than in SubQ (MD, 31.83; adjusted *p* = 0.0011) and RC (MD, 19.56; adjusted *p* = 0.0218) tumors. No significant difference in insulin-positive cell density was detected between SubQ and RC tumors (MD, 12.28; adjusted *p* = 0.1473), reinforcing the distinct neuroendocrine phenotype of OP tumors (Figs. 3U–W and 4D). Secretin expression showed a different trend, with a significantly lower level in SubQ tumors than in either the RC (MD, 18.48; adjusted *p* = 0.0139) or OP (MD, 32.01; adjusted *p* = 0.0004) model. Moreover, no significant difference was detected between tumors of the OP and RC models (MD, 13.52; adjusted *p* = 0.0625) (Figs. 3Z–AB and 4E). Similarly, pancreatic polypeptide expression was significantly higher in tumors in both the RC (MD, 16.77; adjusted *p* = 0.0024) and OP (MD, 27.55; adjusted *p* < 0.0001) models than in tumors in the SubQ model. Moreover, OP tumors exhibited a significant increase in pancreatic polypeptide expression relative to RC tumors (MD, 10.78; adjusted *p* = 0.0304 (Figs. 3AE–AG and 4F). As IHC references, grades 1 and 2 human PNETs exhibited strong immunostaining for all tested markers, including Ki67 (Fig. 3I–J), synaptophysin (Fig. 3N–O), chromogranin A (Fig. 3S–T), insulin (Fig. 3X–Y), secretin (Fig. 3AC–AD), and pancreatic polypeptide (Fig. 3AH–AI), consistent with previously reported PNET characteristics.^38–40^

Finally, we evaluated the contribution of implanted STC-1 cells to tumor formation by measuring EGFP expression at the respective sites of implantation. Significantly higher density of EGFP-positive cells was detected in the OP tumors than in the adjacent benign tissue (MD, 40.76; adjusted *p* = 0.0007). In contrast, no significant difference in EGFP expression was observed between the tumor and adjacent benign tissue in either the SubQ (MD, –0.019; adjusted *p* > 0.9999) or the RC (MD, 16.94; adjusted *p* = 0.3002) model. In addition, the EGFP-positive cell density of the OP tumors was significantly higher than that of the SubQ tumors (MD, 45.49; adjusted *p* = 0.0002) (Figs. 3AJ–AL and 4G), indicating effective engraftment of STC-1 cells in tumors of the OP model.

### Transplanted STC-1 Cells Express PNET Markers in the OP Tumor Model

**Fig. 5:**
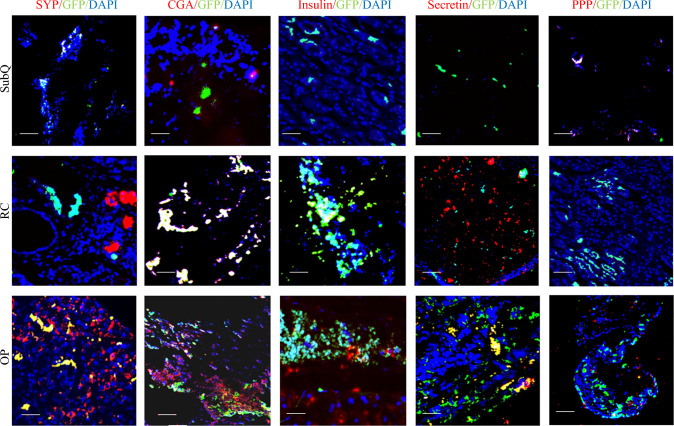
Co-immunofluorescence analysis of neuroendocrine markers in murine pancreatic neuroendocrine tumor (PNET) models. Co-immunofluorescence is shown, performed using neuroendocrine markers (*red*), enhanced green fluorescent protein (EGFP) (*green*), and 4’,6-diamidino-2-phenylindole (DAPI) (*blue*). Cellular proximity was used to assess overlapping, with significant co-localization indicated by yellow and white regions in the images. Immunohistochemical (IHC) staining for synaptophysin (SYP), chromogranin A (CGA), insulin, secretin, and pancreatic polypeptide (PPP) also was performed to evaluate proliferative activity, differentiation, and expression of neuroendocrine marker. EGFP expression was used to track primary tumor cells in the murine PNET models. Scale bar = 200 µm

**Fig. 6:**
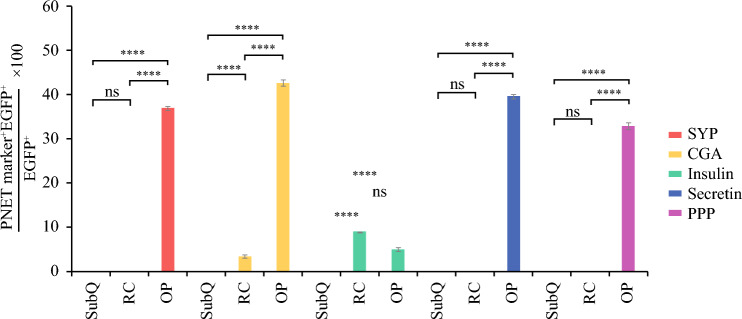
Lineage-tracing of enhanced green fluorescent protein (EGFP)-labeled STC-1 cells in pancreatic neuroendocrine tumor (PNET) models. The localization and differentiation of implanted STC-1 cells within PNETs were assessed by quantifying the proportion of EGFP-positive cells co-expressing PNET markers, including synaptophysin (SYP), chromogranin A (CGA), insulin, secretin, and pancreatic polypeptide (PPP). Comparisons were made across subcutaneous (SubQ), renal capsule (RC), and orthotopic pancreatic (OP) tumor sites. Data were analyzed using one-way ANOVA followed by Tukey’s post hoc test. Statistical significance is indicated as *(*p* < 0.05), **(*p* < 0.01), ***(*p* < 0.001), ****(*p* < 0.0001), and ns (not significant)

To investigate the clonal expansion of injected STC-1 cells and assess their contribution to tumor formation, we performed lineage-tracing studies using co-immunofluorescence labeling of mouse pancreatic neuroendocrine tumor (PNET) samples with EGFP and neuroendocrine-specific markers. The contribution of implanted STC-1 cells to PNET formation was assessed by calculating the proportion of EGFP-positive cells that also expressed PNET markers. In tumors of the OP model, EGFP-positive STC-1 cells exhibited a significantly higher expression of PNET markers, including synaptophysin, chromogranin A, secretin, and pancreatic polypeptide, than in tumors of the SubQ or RC models (Figs. 5A–F, J–O and 6), indicating a critical role of the OP microenvironment in promoting PNET differentiation. Notably, although the effect was less pronounced than that of the OP model, EGFP-positive STC-1 cells in tumors of the RC model did show significantly higher expression of chromogranin A and insulin than those in tumors of the SubQ model, suggesting that the renal microenvironment is more conducive to PNET-like differentiation than the subcutaneous site. Interestingly, although insulin expression in EGFP-positive cells was significantly higher in both OP and RC tumors than in SubQ tumors, less than 10% of EGFP-positive STC1 cells expressed insulin. This suggests that insulin overexpression may not be a major functional feature of the neuroendocrine phenotype in STC-1 cell-derived tumors (Figs. 5D–F, G–I and 6). Collectively, these findings demonstrate that the pancreatic microenvironment most effectively supports PNET development from implanted STC-1 cells. This highlights the robustness of the OP implantation model in recapitulating the neuroendocrine origin of the tumors, thereby providing a valuable platform for investigating the cellular mechanisms underlying PNET progression.

## Discussion

The bioluminescent/fluorescent dual-labeled STC-1 cell line developed in this study represents a valuable tool for PNET research. It enables non-invasive, real-time monitoring of tumor growth in vitro and in vivo across various xenograft models using bioluminescence imaging. Furthermore, the integrated fluorescent marker allows for precise lineage-tracing, facilitating studies on clonal dynamics and the cellular origin of tumors.

In this study, we used the novel dual-labeled STC-1 cell line to develop and compare xenograft PNET models using three commonly used implantation routes: subcutaneous, renal capsule, and OP implantation.^37^ Among the three PNET xenograft models, the OP implantation demonstrated the most consistent tumor engraftment. Moreover, quantification of EGFP-positive cells showed a significantly higher density in tumor tissue than in adjacent non-tumorous regions. These findings, consistent with bioluminescence imaging data, suggest the most successful tumor engraftment and active proliferation within the OP model.

To further validate the STC-1 xenograft models, we evaluated the expression of key human PNET markers. Across all implantation sites, the OP model demonstrated the highest expression of key PNET markers, including synaptophysin, chromogranin A, secretin, and pancreatic polypeptide, compared with both SubQ and RC tumors. Although RC tumors showed slightly higher levels of secretin and insulin than SubQ tumors, the OP site most effectively supported neuroendocrine differentiation and tumor growth. Ki67-staining showed significantly greater proliferation in pancreatic tumors than in either SubQ or RC tumors. This aligns with the increased neuroendocrine marker expression observed in pancreatic tumors, underscoring the pivotal role of the pancreatic microenvironment in promoting PNET progression. Interestingly, one animal in the OP model exhibited metastatic dissemination, indicating that although rare, this model may capture key aspects of tumor progression and metastatic potential. This observation warrants further investigation in future studies.

The lineage-tracing results yielded the interesting finding that the SubQ implantation, although technically straightforward and widely applied in producing xenograft tumor models, showed that significantly less co-localization of EGFP with neuroendocrine markers in SubQ tumors suggests insufficient support for maintaining neuroendocrine identity, likely due to the absence of pancreas-specific stromal and microenvironmental interactions, thereby limiting the model’s utility in studying PNET tumor dynamics.

Unlike the SubQ model, the RC model provided a highly vascularized environment that supports robust tumor engraftment and growth and showed significantly higher co-expression of EGFP with chromogranin A and insulin, confirming successful colonization by neuroendocrine tumor cells. However, the physiologic differences between the kidney and pancreas limit the model’s ability to fully recapitulate pancreas-specific tumor-stroma interactions.

Ultimately, the OP model provided the most physiologically relevant platform for studying PNET progression. It closely mimicked the native tumor microenvironment and supported both robust tumor growth and expression of neuroendocrine markers. Interestingly, co-immunofluorescence analysis of tumors of the OP model showed consistent overlap between EGFP-positive STC-1 cells and all neuroendocrine markers except insulin. Although IHC results showed significantly higher expression of both insulin and EGFP in OP tumors than in RC tumors, the co-immunofluorescence data exhibited no significant difference between the two models. This suggests that the strong IHC signal of insulin in tumors of the OP model is not solely due to the expansion of EGFP-positive STC-1 cells. Given that pancreatic islet *β*-cells also express insulin, we cannot rule out that OP tumor growth may involve or be intermixed with local islet *β* cells. Therefore, the lack of spatial correlation between insulin and EGFP signals suggests that insulin expression is unlikely to represent a primary functional output of STC-1-derived tumors in the OP model. Instead, it points to the presence of heterogeneous mixtures of distinct tumor subtypes. This is not unexpected, given the known heterogeneity of the STC-1 cell line. However, it does make the OP model particularly relevant clinically because it more accurately reflects the inherent heterogeneity of PNETs.

Future studies integrating single-cell RNA sequencing with flow cytometry will be essential to compare the transcriptomic profiles of distinct tumor subpopulations within STC-1-derived OP xenograft tumors. Such analyses could identify key molecular drivers of the PNET phenotype in the OP model and uncover new targets for developing more effective monotherapies or combination therapies, an area already showing promise in PNET treatment.^48^ Overall, these findings emphasize the superiority of the orthotopic model for capturing the complexity of PNET biology and underscore its utility in future mechanistic and preclinical studies.

From a translational standpoint, the choice of in vivo models is critical for enhancing the relevance of preclinical studies and optimizing the evaluation of emerging therapeutic strategies.^41–43^ In the current study, although both the SubQ and RC tumor models offered practical advantages, including technical simplicity and high vascularity, respectively, they failed to replicate the unique pancreatic microenvironment essential for accurately modeling PNET progression and metastasis. In contrast, STC-1 cell-derived OP tumors closely mimicked human PNETs by recapitulating key features. It has been reported that optimal tumor–stroma interactions and pancreas-specific signaling pathways are essential for differentiation and metastasis in pancreatic cancer, as shown in multiple studies.^28,44–46^ The OP model presented in this study is the first PNET mouse xenograft model to integrate these elements, providing a valuable platform for in-depth investigation of the PNET microenvironment. Even more importantly, because STC-1 cells originate from the widely used C57BL/6 background, they are suitable for syngeneic implantation in immunocompetent mice. This enables the convenient development of immune-competent tumor models for evaluating immunotherapies, such as immune checkpoint inhibitors, without relying on technically demanding and costly patient-derived xenograft systems.^19,47^

Compared with the widely used BON-1 and QGP-1 xenografts in NSG mice, the OP model in this study offers a complementary set of strengths and limitations for modeling PNET biology. The STC-1/NuJ system, although constrained by the murine origin of the cell line, retains functional B cells as well as innate immunity that includes functional NK cells, macrophages, and dendritic cells, thereby supporting the investigation of tumor–microenvironment interactions during tumor formation. Consistent with this rationale, our data in the current study demonstrate that the OP model in Nu/J mice more accurately recapitulates the histologic and molecular features of human PNETs than subcutaneous or renal capsule implantation. Future sequencing experiments, including whole-transcriptome RNA-seq analysis, will be performed across the three STC-1 models (SubQ, RC, and OP) to delineate the molecular events underlying STC-1 tumor cell–host interactions that drive PNET development in the OP model.

In contrast to the OP model in this study, BON-1 and QGP-1 xenografts in NSG mice use human-derived PNET cell lines, offering closer molecular relevance to human disease and greater metastatic potential. However, because NSG mice completely lack functional T, B, and NK cells, these models provide limited insight into tumor–host interactions during tumor progression.^48^ Collectively, the OP model described in this study provides a complementary platform to the widely used BON-1 and QGP-1 xenografts in NSG mice, broadening the translational toolkit for PNET research by capturing tumor–host interactions absent in more severely immunodeficient systems.

This study had several limitations that should be considered when the model is applied to future mechanistic or translational investigations. First, although the OP model faithfully recapitulates grade 1 PNET in morphology and neuroendocrine marker expression, its direct translational relevance is constrained by species-specific differences between murine and human tumor biology.^49^

Second, although the model reliably reproduces well-differentiated PNET, it does not recapitulate advanced or metastatic disease, with metastasis observed only in a single animal, thereby limiting its use for evaluating therapies aimed at disseminated PNET. This limitation is consistent with the known challenges of cell line-derived xenografts, which often fail to model the metastatic cascade, underscoring the importance of complementary systems such as GEMMs or PDXs for studying late-stage disease.^50,51^

Finally, because the OP model was established in immunodeficient Nu/J mice, immune-mediated mechanisms cannot be evaluated. Therefore, future studies will focus on adapting the OP implantation of STC-1 cells to generate syngeneic, immunocompetent PNET models, enabling investigation of PNET–immune interactions and supporting the development of novel immunotherapies.^52^

In conclusion, the STC-1 OP model in this study offers a novel opportunity to study PNET tumor growth in biologically relevant mouse models and provides a pragmatic preclinical tool for investigating the effects of novel therapies, either alone or in combination with other established PNET treatments.^21^

## Supplementary Information

Below is the link to the electronic supplementary material.

Supplementary file1 (DOCX 19 kb)

## Data Availability

Additional data supporting the conclusions of this study are available from the corresponding author upon request through Cooper University Hospital. Access to the data is subject to restrictions in accordance with HIPAA guidelines to ensure patient confidentiality.
