# Supplementary material for: Lighting up PNETs: Creating Murine Models with a Novel Bioluminescent Cell Line
Source: Ann Surg Oncol. 2026 Feb 3;33(5):4376–87. doi: 10.1245/s10434-026-19089-z (PMC13083327; doi:10.1245/s10434-026-19089-z)
Supplement: Supplementary file 1 — Supplementary file1 (DOCX 19 kb) [file 10434_2026_19089_MOESM1_ESM.docx]

**TABLE S1** Results of two-way ANOVA test of postoperative weight over time

| Table analyzed | Mouse weight over time | | | | | | | |
| --- | --- | --- | --- | --- | --- | --- | --- | --- |
| Two-way ANOVA | Ordinary |  |  |  |  |  |  |  |
| Alpha | 0.05 |  |  |  |  |  |  |  |
| Source of variation | % of total variation | *p* Value | *p* Value summary | Significant? |  |  |  |  |
| Interaction | 8.853 | 0.0015 | ** | Yes |  |  |  |  |
| Row factor | 33.98 | <0.0001 | **** | Yes |  |  |  |  |
| Column factor | 42.38 | <0.0001 | **** | Yes |  |  |  |  |
| ANOVA table | SS | DF | MS | F(DFn, DFd) | *p* Value |  |  |  |
| Interaction | 66.66 | 12 | 5.555 | F(12, 63) = 3.142 | 0.0015 |  |  |  |
| Row factor | 255.8 | 6 | 42.64 | F(6, 63) = 24.12 | 0.0001 |  |  |  |
| Column factor | 319.1 | 2 | 159.6 | F(2, 63) = 90.24 | 0.0001 |  |  |  |
| Residual | 111.4 | 63 | 1.768 |  |  |  |  |  |
| Data summary | | | | | | | | |
| No. of columns (column factor) | 3 |  |  |  |  |  |  |  |
| No. of rows (row factor) | 7 |  |  |  |  |  |  |  |
| No. of values | 84 |  |  |  |  |  |  |  |
| Within each row, compare columns (simple effects within rows) | | | | | | | | |
| No. of families | 7 |  |  |  |  |  |  |  |
| No. of comparisons per family | 3 |  |  |  |  |  |  |  |
| Alpha | 0.05 |  |  |  |  |  |  |  |
| Tukey's multiple comparisons test | Mean difference | 95.00 % CI of difference | Below threshold? | Summary^a^ | Adjusted *p* Value |  |  |  |
| PreOp | | | | | | | | |
| SubQ vs RC | 0.75 | –1.507 to 3.007 | No | NS | 0.7058 |  |  |  |
| SubQ vs OP | 0.175 | –2.082 to 2.432 | No | NS | 0.9811 |  |  |  |
| RC vs OP | –0.575 | –2.832 to 1.682 | No | NS | 0.8144 |  |  |  |
| Week 1 | | | | | | | | |
| SubQ vs RC | 2.5 | 0.2431 to 4.757 | Yes | * | 0.0265 |  |  |  |
| SubQ vs OP | 3.25 | 0.9931 to 5.507 | Yes | ** | 0.0028 |  |  |  |
| RC vs OP | 0.75 | –1.507 to 3.007 | No | NS | 0.7058 |  |  |  |
| Week 2 | | | | | | | | |
| SubQ vs RC | 4.825 | 2.568 to 7.082 | Yes | **** | <0.0001 |  |  |  |
| SubQ vs OP | 6.15 | 3.893 to 8.407 | Yes | **** | <0.0001 |  |  |  |
| RC vs OP | 1.325 | –0.9319 to 3.582 | No | NS | 0.3423 |  |  |  |
| Week 3 | | | | | | | | |
| SubQ vs RC | 4.85 | 2.593 to 7.107 | Yes | **** | <0.0001 |  |  |  |
| SubQ vs OP | 6.5 | 4.243 to 8.757 | Yes | **** | <0.0001 |  |  |  |
| RC vs OP | 1.65 | –0.6069 to 3.907 | No | NS | 0.1932 |  |  |  |
| Week 4 | | | | | | | | |
| SubQ vs RC | 4.7 | 2.443 to 6.957 | Yes | **** | <0.0001 |  |  |  |
| SubQ vs OP | 4.9 | 2.643 to 7.157 | Yes | **** | <0.0001 |  |  |  |
| RC vs OP | 0.2 | –2.057 to 2.457 | No | NS | 0.9754 |  |  |  |
| Week 5 | | | | | | | | |
| SubQ vs RC | 5.5 | 3.243 to 7.757 | Yes | **** | <0.0001 |  |  |  |
| SubQ vs OP | 5.25 | 2.993 to 7.507 | Yes | **** | <0.0001 |  |  |  |
| RC vs OP | –0.25 | –2.507 to 2.007 | No | NS | 0.9618 |  |  |  |
| Week 6 | | | | | | | | |
| SubQ vs RC | 4.5 | 2.243 to 6.757 | Yes | **** | <0.0001 |  |  |  |
| SubQ vs OP | 3.875 | 1.618 to 6.132 | Yes | *** | 0.0003 |  |  |  |
| RC vs OP | –0.625 | –2.882 to 1.632 | No | NS | 0.7847 |  |  |  |
| Test details | Mean 1 | Mean 2 | Mean difference | SE of difference | N1 | N2 | Q | DF |
| PreOp | | | | | | | | |
| SubQ vs RC | 23.5 | 22.75 | 0.75 | 0.9402 | 4 | 4 | 1.128 | 63 |
| SubQ vs OP | 23.5 | 23.33 | 0.175 | 0.9402 | 4 | 4 | 0.2632 | 63 |
| RC vs OP | 22.75 | 23.33 | –0.575 | 0.9402 | 4 | 4 | 0.8649 | 63 |
| Week 1 | | | | | | | | |
| SubQ vs RC | 24 | 21.5 | 2.5 | 0.9402 | 4 | 4 | 3.76 | 63 |
| SubQ vs OP | 24 | 20.75 | 3.25 | 0.9402 | 4 | 4 | 4.888 | 63 |
| RC vs OP | 21.5 | 20.75 | 0.75 | 0.9402 | 4 | 4 | 1.128 | 63 |
| Week 2 | | | | | | | | |
| SubQ vs RC | 25.5 | 20.68 | 4.825 | 0.9402 | 4 | 4 | 7.257 | 63 |
| SubQ vs OP | 25.5 | 19.35 | 6.15 | 0.9402 | 4 | 4 | 9.25 | 63 |
| RC vs OP | 20.68 | 19.35 | 1.325 | 0.9402 | 4 | 4 | 1.993 | 63 |
| Week 3 | | | | | | | | |
| SubQ vs RC | 24.25 | 19.4 | 4.85 | 0.9402 | 4 | 4 | 7.295 | 63 |
| SubQ vs OP | 24.25 | 17.75 | 6.5 | 0.9402 | 4 | 4 | 9.777 | 63 |
| RC vs OP | 19.4 | 17.75 | 1.65 | 0.9402 | 4 | 4 | 2.482 | 63 |
| Week 4 | | | | | | | | |
| SubQ vs RC | 22.5 | 17.8 | 4.7 | 0.9402 | 4 | 4 | 7.069 | 63 |
| SubQ vs OP | 22.5 | 17.6 | 4.9 | 0.9402 | 4 | 4 | 7.37 | 63 |
| RC vs OP | 17.8 | 17.6 | 0.2 | 0.9402 | 4 | 4 | 0.3008 | 63 |
| Week 5 | | | | | | | | |
| SubQ vs RC | 22.5 | 17 | 5.5 | 0.9402 | 4 | 4 | 8.273 | 63 |
| SubQ vs OP | 22.5 | 17.25 | 5.25 | 0.9402 | 4 | 4 | 7.897 | 63 |
| RC vs OP | 17 | 17.25 | –0.25 | 0.9402 | 4 | 4 | 0.376 | 63 |
| Week 6 | | | | | | | | |
| SubQ vs RC | 20.88 | 16.38 | 4.5 | 0.9402 | 4 | 4 | 6.768 | 63 |
| SubQ vs OP | 20.88 | 17 | 3.875 | 0.9402 | 4 | 4 | 5.828 | 63 |
| RC vs OP | 16.38 | 17 | –0.625 | 0.9402 | 4 | 4 | 0.9401 | 63 |

ANOVA, analysis of variance; SS, ; DS, ; MS, ; DFn, ; DFd, ; CI, confidence interval; PreOP, preoperative; SubQ, subcutaneous; RC, renal capsule; OP, orthotopic pancreas; SE, ; Q, ; DF,<AQ8>

^a^Statistical significance is indicated as *(*p* < 0.05) , **(*p* < 0.01), ***(*p* < 0.001), ****(*p* < 0.0001), and NS (not significant).
